# The molecular mechanism underlying dermatomyositis related interstitial lung disease: evidence from bioinformatic analysis and *in vivo* validation

**DOI:** 10.3389/fimmu.2023.1288098

**Published:** 2023-10-19

**Authors:** Li Zeng, Yiping Tang, Yichen Zhang, Li Yue, Gang Ma, Xumin Ye, Lijing Yang, Kai Chen, Qiao Zhou

**Affiliations:** ^1^ Department of Neurology, Sichuan Academy of Medical Science and Sichuan Provincial People’s Hospital, University of Electronic Science and Technology of China, Chengdu, China; ^2^ Department of Internal Medicine, Sichuan Academy of Medical Science and Sichuan Provincial People’s Hospital, University of Electronic Science and Technology of China, Chengdu, China; ^3^ Department of Rheumatology and Immunology, Sichuan Academy of Medical Science and Sichuan Provincial People’s Hospital, University of Electronic Science and Technology of China, Chengdu, China; ^4^ School of Medicine, University of Electronic Science and Technology of China, Chengdu, China; ^5^ Chengdu University of Traditional Chinese Medicine, Chengdu, China; ^6^ Southwest Medical University, Luzhou, China; ^7^ Clinical Immunology Translational Medicine Key Laboratory of Sichuan Province, Sichuan Provincial People’s Hospital, University of Electronic Science and Technology of China, Chengdu, China

**Keywords:** dermatomyositis, interstitial lung disease, idiopathic pulmonary fibrosis, bioinformatic analysis, extracellular matrix

## Abstract

**Background:**

Dermatomyositis (DM) is an autoimmune and inflammatory disease that can affect the lungs, causing interstitial lung diseases (ILD). However, the exact pathophysiological mechanisms underlying DM-ILD are unknown. Idiopathic pulmonary fibrosis (IPF) belongs to the broader spectrum of ILD and evidence shows that common pathologic pathways might lie between IPF and DM-ILD.

**Methods:**

We retrieved gene expression profiles of DM and IPF from the Gene Expression Omnibus (GEO) and utilized weighted gene co-expression network analysis (WGCNA) to reveal their co-expression modules. We then performed a differentially expressed gene (DEG) analysis to identify common DEGs. Enrichment analyses were employed to uncover the hidden biological pathways. Additionally, we conducted protein-protein interaction (PPI) networks analysis, cluster analysis, and successfully found the hub genes, whose levels were further validated in DM-ILD patients. We also examined the relationship between hub genes and immune cell abundance in DM and IPF. Finally, we conducted a common transcription factors (TFs)-genes network by NetworkAnalyst.

**Results:**

WGCNA revealed 258 intersecting genes, while DEG analysis identified 66 shared genes in DM and IPF. All of these genes were closely related to extracellular matrix and structure, cell-substrate adhesion, and collagen metabolism. Four hub genes (*POSTN*, *THBS2*, *COL6A1*, and *LOXL1*) were derived through intersecting the top 30 genes of the WGCNA and DEG sets. They were validated as active transcripts and showed diagnostic values for DM and IPF. However, ssGSEA revealed distinct infiltration patterns in DM and IPF. These four genes all showed a positive correlation with immune cells abundance in DM, but not in IPF. Finally, we identified one possible key transcription factor, MYC, that interact with all four hub genes.

**Conclusion:**

Through bioinformatics analysis, we identified common hub genes and shared molecular pathways underlying DM and IPF, which provides valuable insights into the intricate mechanisms of these diseases and offers potential targets for diagnostic and therapeutic interventions.

## 1 Introduction

Interstitial lung diseases (ILD) are a group of diseases characterized by inflammation and fibrosis in the lung tissue, leading to difficulty in breathing and reduced lung function (1). ILD encompasses heterogeneous disorders, including idiopathic pulmonary fibrosis (IPF) and connective tissue disease associated ILD (CTD-ILD), etc (2–7). IPF (with no known cause) is the most common and severe subtype of ILD, and it is characterized by excessive fibrous tissue growth in the lungs which impairs lung function and may lead to respiratory failure (8). Dermatomyositis (DM) is a rare CTD that primarily affects the skin and muscles (2). However, it can also involve other organs, with ILD being the most common extramuscular manifestation. The prevalence of ILD in DM is significantly higher, ranging from approximately 20% to 80%, compared to the general population (9–11). The clinical courses and prognosis of DM-ILD can vary greatly and sometimes it is more challenging to treat and has a worse prognosis compared to other myositis-related ILD (12, 13). Recent studies have highlighted the importance of assessing myositis-specific autoantibodies (MSAs) which are in relation to the clinical phenotypes of DM-ILD (14, 15). However, the exact pathophysiological mechanisms underlying DM-ILD is still unknown.

Signaling pathways such as transforming growth factor -β (TGF-β), WNT, hedgehog, and platelet-derived growth factor (PDGF) have been linked to the development IPF and some other ILD (16). These inflammatory pathways can activate fibroblasts and stimulate their differentiation into myofibroblasts, which then produce large amounts of extracellular matrix (ECM). The excessive ECM deposition leads to the continuous restructuring of pulmonary tissue, causing fibrosis and the development of scar tissue (17). No disparities were observed in PDGF, FGF-2, and VEGF levels among IPF or other ILD patients, as indicated by a study conducted on lung explants (18). The FDA granted approval to nintedanib in March 2020 for the treatment of progressive fibrosing ILD in all patients regardless of the cause based on the results from INBUILD trial (19). Considering the clinical and pathophysiological similarities among IPF and some disease associated ILD, it has been suggested that such disorders might have shared pathologic pathways (20).

**Figure 1:**
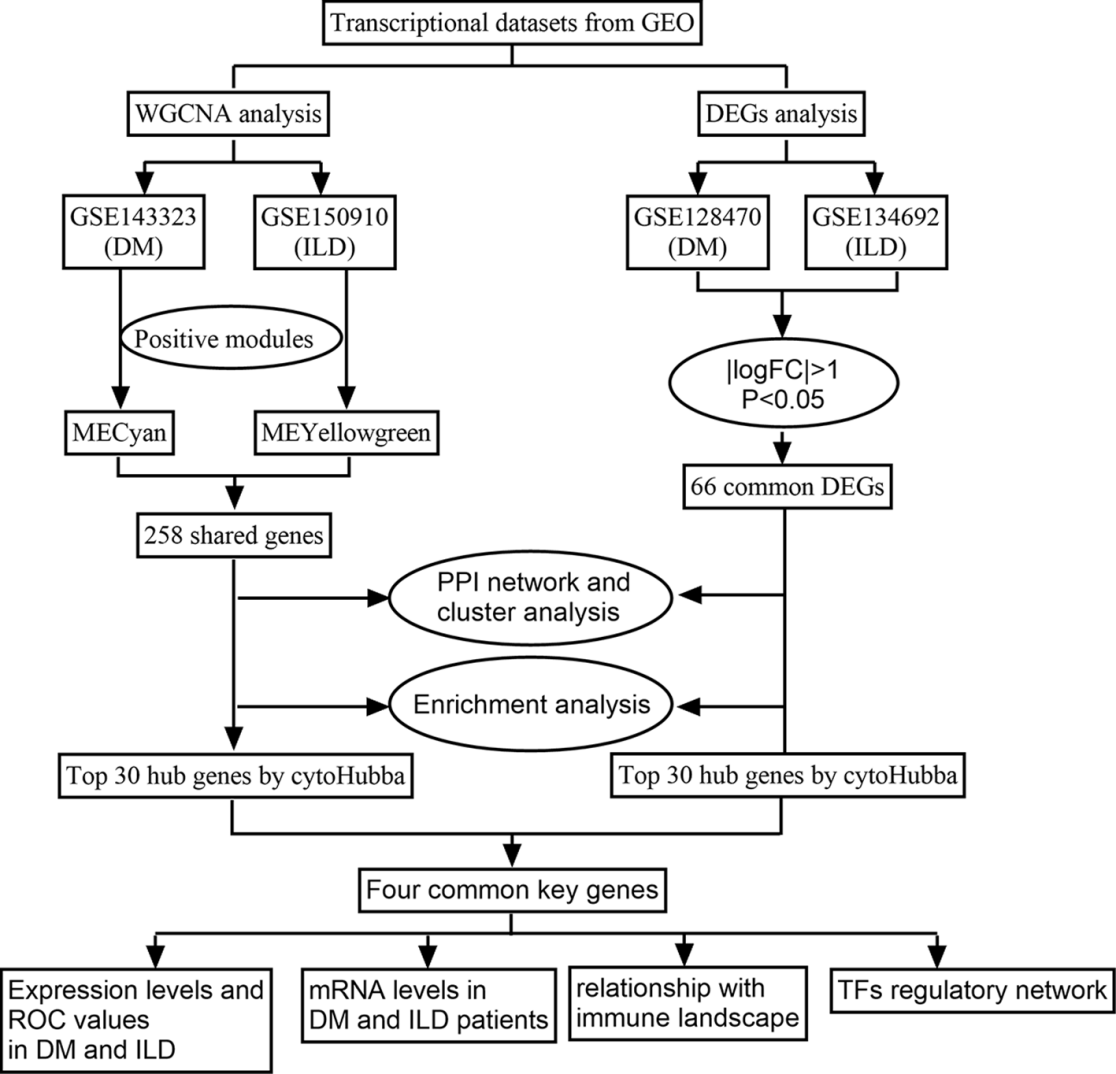
The flowchart for bioinformatic analysis.

By analyzing the genetic basis and shared molecular pathways between IPF and DM, we may gain insight into the mechanisms in the development of DM-ILD. In this study, we aim to utilized gene expression profiles from public databases to identify co-expression modules associated with both IPF and DM. Furthermore, we conducted an analysis of differentially expressed genes (DEGs) in order to identify the shared genes in both diseases in separate datasets. Additionally, we employed protein protein interaction (PPI) and cluster analysis to uncover the common hub genes and investigated the relationship between hub gene and immune landscape in both IPF and DM. The research flowchart is depicted in **Figure 1**. By revealing these shared gene signatures and molecular pathways, we hope to enhance our comprehension of the pathogenesis of DM-ILD and indicate potential treatment targets to improve outcomes for affected patients.

## 2 Materials and methods

### 2.1 Data source

We retrieved four datasets from the Gene Expression Omnibus (GEO) database, including DM datasets GSE143323 and GSE128470, as well as IPF datasets GSE150910 and GSE134692. The detailed information of these datasets can be found in **Supplementary File 1**. For subsequent bioinformatic analysis, GSE143323 and GSE150910 were matched for the WGCNA analysis, while GSE128470 and GSE134692 were matched for DEG analysis.

### 2.2 WGCNA construction and identification of common disease related module genes in DM and IPF

The co-expression network construction in the GSE143323 and GSE150910 datasets was performed using the WGCNA package in R (21). Initially, the variance of expression values for each gene was calculated. Genes were filtered out if their absolute deviations were higher than 25% of the median. Subsequently, the goodSampleGenes function was employed to exclude the outlier samples, resulting in the exclusion of three samples from GSE143323 and one sample from GSE150910 due to high heterogeneity (**Supplementary Files 2A, B**). The determination of a proper soft threshold for constructing a scale-free network was accomplished using the “pickSoftThreshold” function, which yielded a soft threshold of 22 in DM and 6 in IPF (**Supplementary Files 2C, D**). A hierarchical clustering dendrogram was then built to categorize similar genes into modules each containing a minimum of 30 genes. These modules were further consolidated based on a module eigengenes dissimilarity threshold (MEDissThres) of 0.4. Finally, Pearson correlation analysis was used to evaluate the relationship between each module and diseases. The modules exhibiting the strongest positive correlations with DM or IPF were chosen. 258 intersecting genes were obtained from these modules through the Jvenn online tool at http://jvenn.toulouse.inra.fr/app/example.html (22).

### 2.3 Identification of common DEGs in DM and IPF

The DEG analysis in the GSE128470 and GSE134692 datasets was conducted using the “limma” package in R. The screening criteria for significant differential expression were defined as log2|fold change (FC)|>1 and *P* value<0.05. A total of 66 common DEGs were obtained from DM and IPF datasets.

### 2.4 PPI network, cluster analysis and enrichment analysis

The PPI network was constructed using the STRING online database (the Search Tool for Retrieval of Interacting Genes; http://string-db.org). Cytoscape (V3.7.2) was employed to visualize the PPI networks, with an interaction score of at least 0.4. Next, cluster analysis was done using the MCODE algorithm, a plug-in of Cytoscape, focusing on gene clusters with scores > 5. Biological functions and pathways of the common genes were conducted using Gene Ontology (GO) and Kyoto Encyclopedia of Genes and Genomes (KEGG) analyses in R with the “enrichplot” and “clusterProfiler” packages. Terms and pathways with *P* value less than 0.05 were considered significant statistically.

### 2.5 Identification and validation of Common hub genes

Maximal clique centrality (MCC) algorithm, a plug-in of cytoHubba, was utilized to identify the hub genes in the PPI network. Through intersecting the top 30 genes obtained separately from WGCNA and DEG, four hub genes were identified. Subsequently, their expression levels were validated in each of the four datasets using GraphPad Prism (V9.3). The diagnostic powers of these genes were calculated by creating receiver operating characteristic (ROC) curves with the R package “pROC”.

### 2.6 Reverse transcription-quantitative polymerase chain reaction (RT-PCR) analysis

Between 2021 and 2022, a total of 9 adult inpatients with active DM-ILD were included in our study. The DM diagnoses were established based on the Bohan and Peter criteria and was confirmed through a skin/muscle biopsy (23). Patients were considered with ILD if they met the following: (i) they had a restrictive impairment in pulmonary function, with total lung capacity (TLC) and diffusion capacity for carbon monoxide of the lung (DLCO) both being less than 80% of the predicted values, and (ii) their high-resolution computed tomography (HRCT) results displayed signs like reticulonodular, nodular, linear or ground-glass opacities, consolidations, irregular interface, honeycombing, or traction bronchiectasis. Patients combined with infectious disease, metabolic diseases, other autoimmune disease, or cancer, etc were excluded. As control subjects, 11 healthy participants undergoing routine health examinations were included as health controls. For each subject, 1 ml blood was taken. Our study was carried out in compliance with the Declaration of Helsinki and received approval from the Ethical Committee of our hospital. Additionally, all participants provided their informed consent by signing the necessary forms. Total RNA from whole blood was isolated using TransZol Up Plus RNA Kit (TransGen Biotech, Beijing) and reverse transcribed (TransScript All-in-One Kit, TransGen Biotech, Beijing). The PCR analysis was done on an ABI StepOnePlus™ system using TransStart Top Green qPCR SuperMix (TransGen Biotech, Beijing) in triplicate. The 2^–△△Ct^ method was used to calculate the relative expressions of the target genes, with GAPDH as the housekeeping gene. All the sequences of the primers are listed in **Supplementary File 3**.

### 2.7 Single-sample gene set enrichment analysis (ssGSEA)

The immune infiltration pattern in both DM and IPF patients were assessed by the ssGSEA algorithm in R package (23). The expression levels of the infiltrating immune cells were represented using boxplots. Furthermore, a Spearman correlation analysis was performed using the “ggplot2” package to examine the correlation between the hub genes and the immune cells.

### 2.8 Identification of Transcription factors (TFs)-genes network

Interactions between the TFs and the hub genes were analyzed using the NetworkAnalyst tool (V2019; https://www.networkanalyst.ca/) and visualized with Cytoscape.

## 3 Results

### 3.1 Identification of common gene signatures in DM and IPF by WGCNA

**Figure 2:**
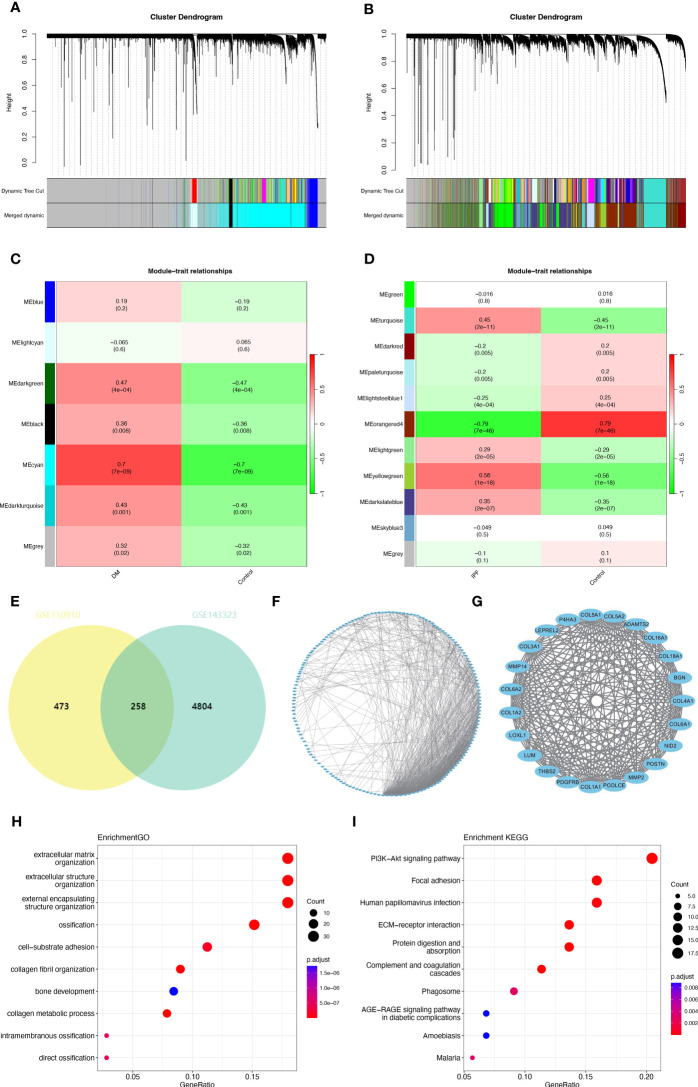
Common gene signatures in GSE143323 (DM) and GSE150910 (IPF) datasets using WGCNA algorithm. **(A, B)** The Cluster dendrogram in GSE143323 (DM) and in GSE150910 (IPF). **(C, D)** Heatmap illustrating the module-trait relationships in GSE143323 (DM) and GSE 150910 (IPF). **(E)** The overlapped genes between the cyan module in GSE143323 (DM) and the yellowgreen module in GSE150910 (IPF). **(F, G)** A PPI network for the 258 common genes and one cluster extracted using MCODE. **(H, I)** Top 10 GO and KEGG pathways associated with the cluster. DM, dermatomyositis; GO, Gene Ontology; IPF, idiopathic pulmonary fibrosis; KEGG, Kyoto Encyclopedia of Genes and Genomes; MCODE, Minimal Common Oncology Data Elements; PPI, protein-protein interaction; WGCNA, weighted gene coexpression network analysis.

In the GSE143323 dataset, seven modules were screened out through WGCNA, each displayed with a distinct color. Subsequently, the relationship between each disease and modules was evaluated using a Spearman correlation coefficient heatmap (**Figures 2A, C**). Notably, the module “MEcyan” exhibited the strongest correlation with DM and was therefore designated as the DM-related module (r = 0.7, p = 7e-09).

Similarly, in the GSE150910 dataset, eleven modules were obtained through WGCNA. Among them, two modules, “Meyellowgreen” and “Meorangered4,” demonstrated the highest positive and negative correlations with IPF, respectively (Meyellowgreen: r = 0.56, p = 1e-18; Meorangered4: r = -0.79, p = 7e-46) (**Figures 2B, D**). To ensure consistency with GSE143323, the module “Meyellowgreen,” encompassing 731 genes, was designated as the IPF-related module (**Figure 2D**). Totally, 258 common genes were selected out by intersecting the gene modules positively related to DM (Mecyan) and IPF (Meyellowgreen) (**Figure 2E**). Next, a PPI network of these genes was constructed using STRING. After excluding the individual proteins, a network containing 184 nodes and 810 links was obtained (**Figure 2F**). Subsequently, the MCODE plug-in was employed to extract one closely connected gene cluster module (**Figure 2G**). To explore these genes’ possible biological functions, functional enrichment analysis was performed. GO analysis indicated that they were primarily associated with ECM and structure, cell-substrate adhesion, and collagen metabolism (**Figure 2H**). Additionally, according to KEGG analysis, this gene set exhibited strong enrichment in the phosphoinositide-3-kinase-protein kinase B/Akt (PI3K-Akt) signaling pathway, ECM-receptor interaction, and focal adhesion (**Figure 2I**).

### 3.2 Verification of common DEGs in DM and IPF

**Figure 3:**
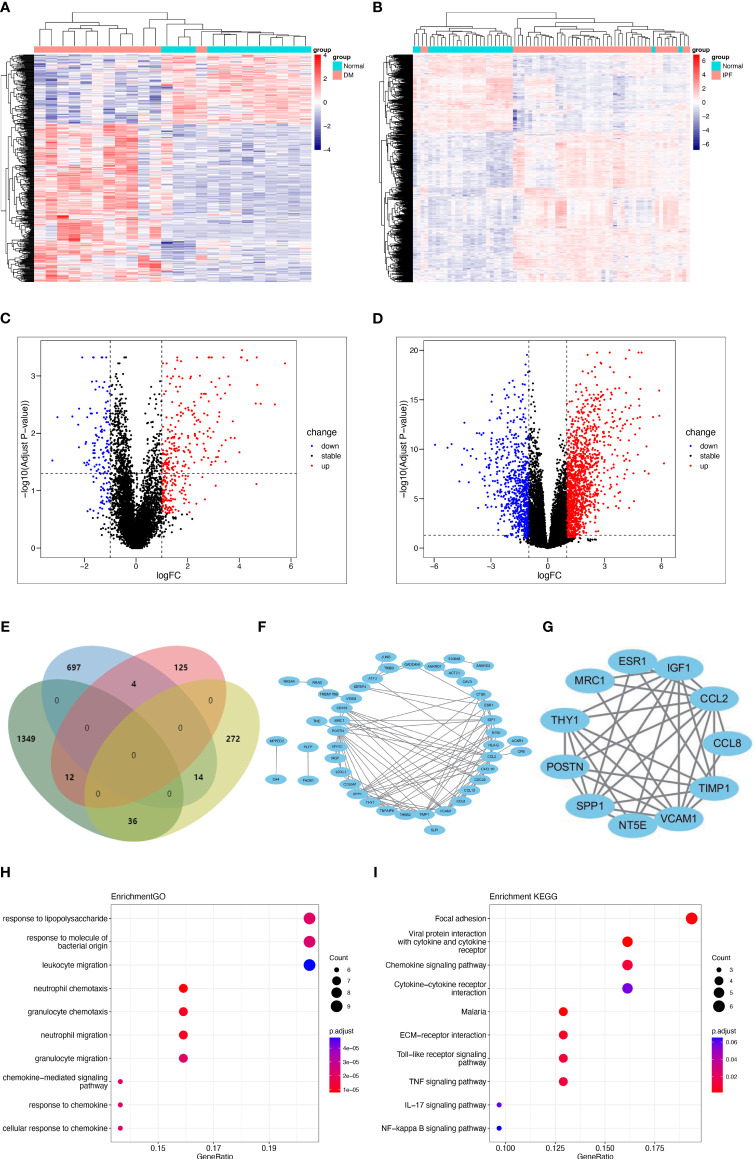
Shared DEGs in DM and IPF and functional analysis. **(A, B)** Heatmap of DEGs in GSE128470 (DM) and GSE134692 (IPF) datasets. **(C, D)** Volcano plots illustrating DEGs in GSE128470 (DM) and GSE134692 (IPF) datasets. **(E)** Venn diagram illustrating the 66 intersecting DEGs in DM and IPF datasets. **(F, G)** A PPI network for the 66 shared DEGs and one cluster was extracted from MCODE. **(H, I)** Top 10 GO and KEGG terms associated with the shared DEGs. DEGs, differentially expressed genes; DM, dermatomyositis; GO, Gene Ontology; KEGG, Kyoto Encyclopedia of Genes and Genomes; MCODE, Minimal Common Oncology Data Elements; IPF, idiopathic pulmonary fibrosis; PPI, protein-protein interaction.

To validate our findings, DEG analysis was performed on GSE128470 and GSE134692 datasets. In GSE128470, 463 DEGs (322 upregulated and 141 downregulated genes) were found, while GSE134692 revealed 2109 DEGs (1397 upregulated and 712 downregulated genes). Heatmaps and Volcano plots were utilized to visualize these DEGs (**Figures 3A–D**). Venn diagram indicated that there were 66 genes common to both datasets (36 common upregulated, 6 common down-regulated and 26 genes with inconsistent expression) (**Figure 3E**). A PPI network of these genes was also constructed, resulting in 45 nodes and 111 links after the exclusion of individual ones (**Figure 3F**). Subsequently, an 11-node and 35-link cluster was extracted using the MCODE plug-in (**Figure 3G**). Similar GO or KEGG enrichment was performed for these DEGs. GO analysis revealed that the four genes were predominantly associated with neutrophil/granulocyte migration, neutrophil and granulocyte chemotaxis (**Figure 3H**). KEGG analysis showed that they significantly enriched in focal adhesion, chemokine signaling pathway, and ECM-receptor interaction (**Figure 3I**). Interestingly, in accordance with the WGCNA results, “ECM-receptor interaction” and “focal adhesion” were enriched once again.

### 3.3 Selection and validation of common hub gene

**Figure 4:**
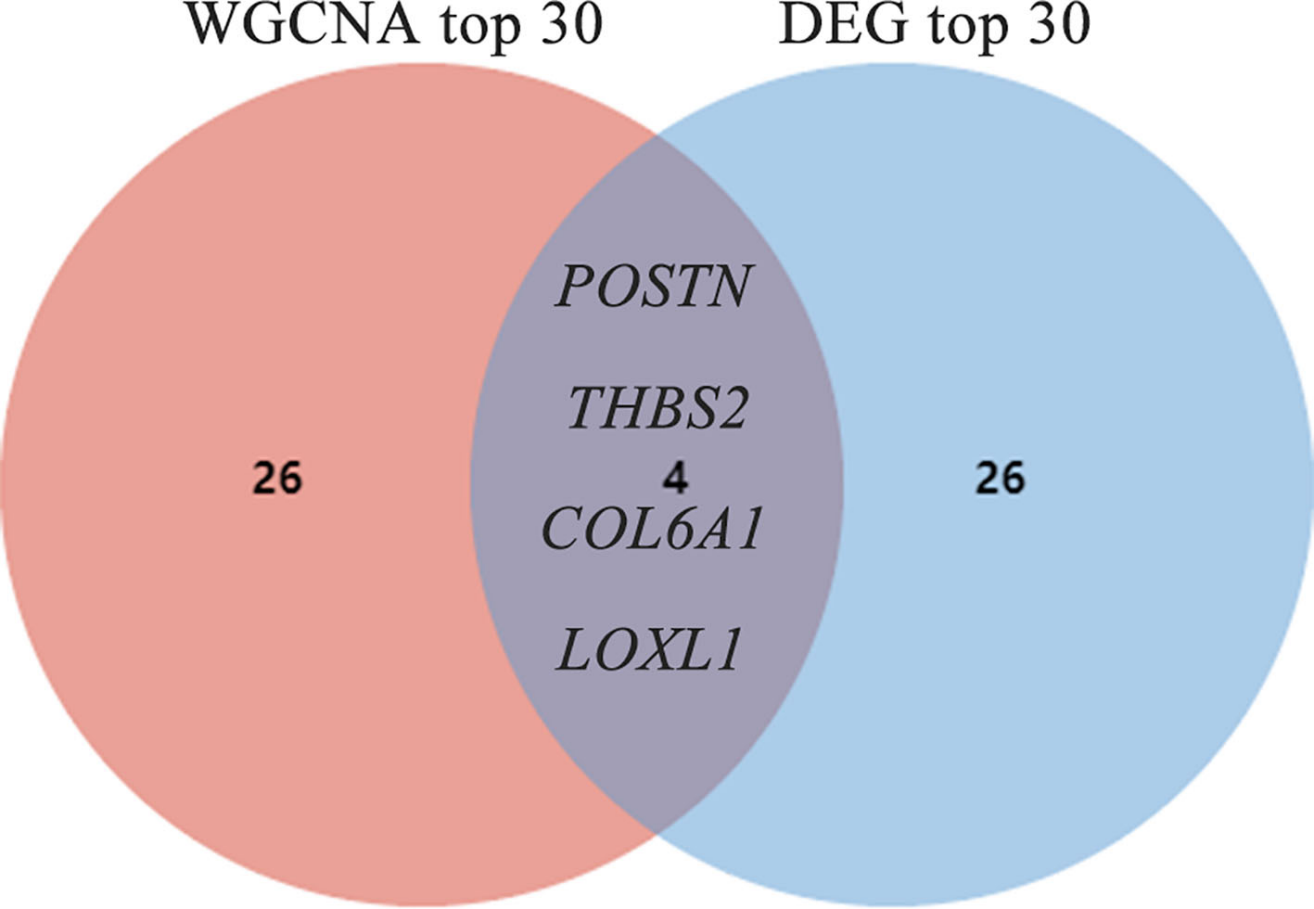
Screening out of common hub genes through the intersection of the top 30 genes derived from DEG and WGCNA. DEGs, differentially expressed genes; WGCNA, weighted gene coexpression network analysis;.

**Figure 5:**
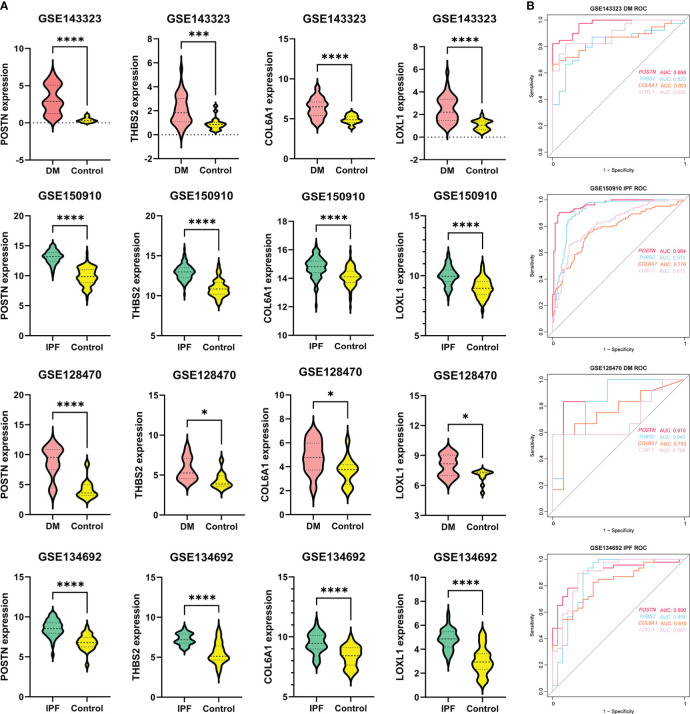
The expression levels and diagnostic efficacy of the common genes. **(A)** *POSTN*, *THBS2*, *COL6A1* and *LOXL1* expression levels in four independent databases, with red violin plots representing DM, blue representing IPF and yellow representing controls. Students’ t-test with p < 0.05 was used to determine statistical significance. *p<0.05; ***p<0.001; ****p<0.0001. **(B)** The ROC curves showing AUC values of *POSTN*, *THBS2*, *COL6A1* and *LOXL1* in DM and IPF. DM, dermatomyositis; IPF, idiopathic pulmonary fibrosis; AUC, area under curve.

**Figure 6:**
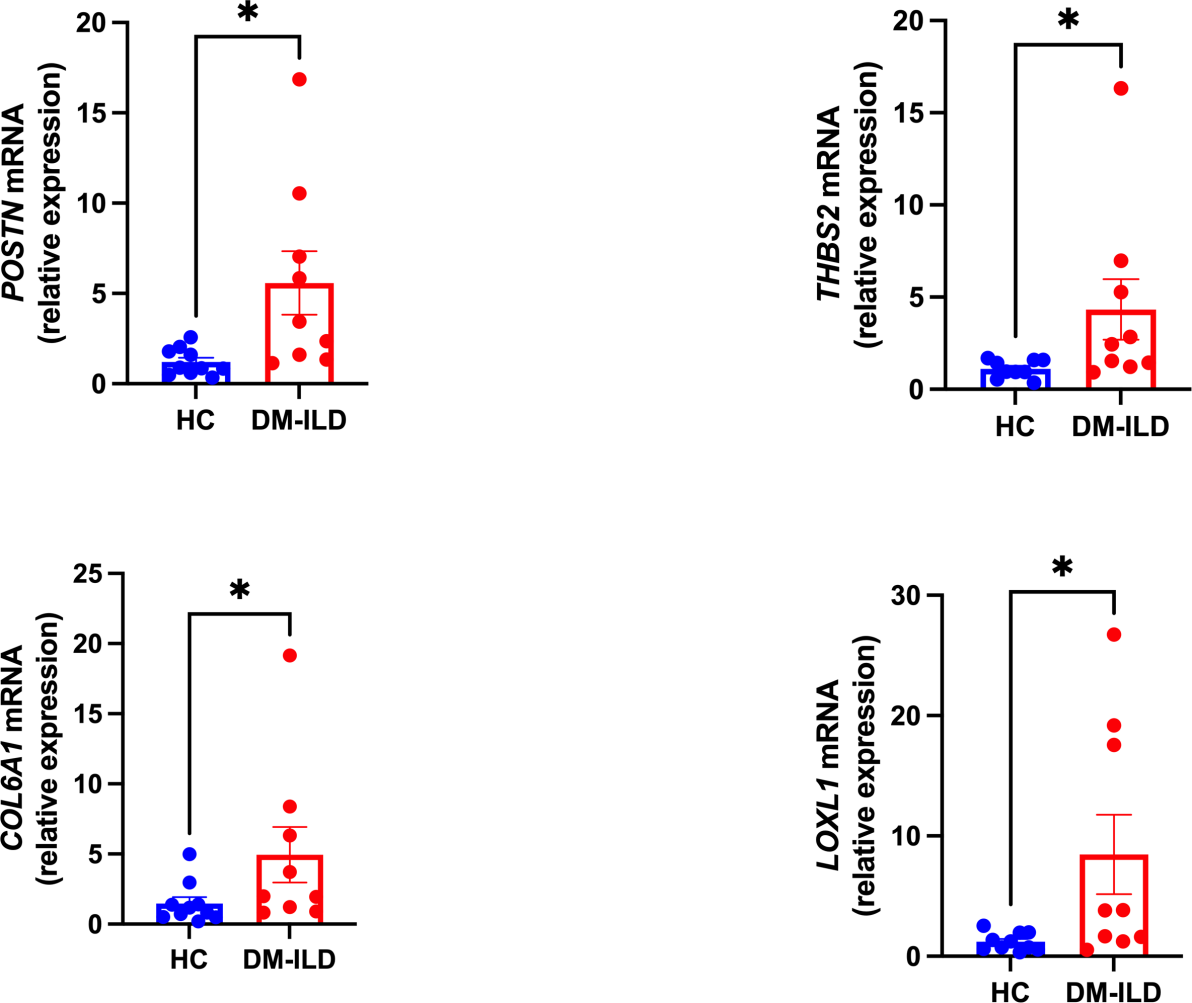
Transcription levels of the *POSTN*, *THBS2*, *COL6A1*, and *LOXL1* mRNA were analyzed in DM-ILD patients (n=9) as well as in health controls (n=10). A nonparametric Student’s t test was calculated when comparing the two groups. *P* value less than 0.05 was considered significant statistically. *p<0.05.

Shared hub genes were identified through PPI networks analysis using cytoHubba, a Cytoscape plug-in (24). By employing the MCC algorithm, the top 30 genes were recognized as potential hubs. After intersecting the top 30 ones from the WGCNA and DEG datasets, four hub genes (*POSTN*, *THBS2*, *COL6A1*, and *LOXL1*) were identified (**Figure 4**). Their expression levels were subsequently validated in the four datasets. It is interesting to note the elevated expression of all genes in both DM and IPF, compared to control group (**Figure 5A**). Moreover, the diagnostic efficacy of these four genes was also evaluated in the four datasets. All four genes demonstrated substantial diagnostic value in both DM and IPF, particularly *POSTN* and *THBS2*, both showing area under the curve (AUC) values exceeding 0.8 (**Figure 5B**). Further quantification of the mRNA abundance of these four genes revealed that they were all actively transcribed in the whole blood of DM-ILD patients (**Figure 6**).

### 3.4 Comparison of immune infiltration microenvironment of DM and IPF

**Figure 7:**
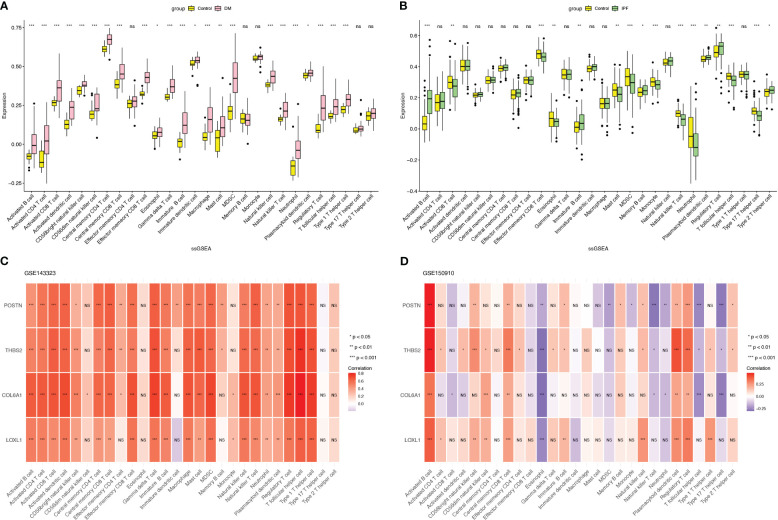
Assessment of immune infiltration microenvironment in DM and IPF with the ssGSEA algorithm. **(A, B)** Boxplot displaying the distribution of immune cells in DM and IPF. **(C, D)** The relationship of common hub genes and 28 types of immune cells in DM and IPF. ns=non-significant; *p< 0.05; **p< 0.01; ***p< 0.001. DM, dermatomyositis; IPF, idiopathic pulmonary fibrosis.

To explore the shared pathogenic mechanisms and immune microenvironments between DM and IPF, we applied the ssGSEA algorithm to comprehensively assess the extent of immune cell infiltration in the two diseases. Unexpectedly, the result diverged from the initial hypothesis, as DM and IPF exhibited significantly disparate immune cell infiltration patterns. As shown in **Figure 7A**, various immune cell types demonstrated significant activation in DM, including B cells, T cells, dendritic cells (DCs), macrophages, and natural killer (NK) cells. In contrast, IPF demonstrated restricted immune cell activation, as evidenced by the existence of a limited number of significantly activated immune cells, including activated B cells, effector memory CD8 T cells, myeloid-derived suppressor cells (MDSCs), monocytes, natural killer T cells, T follicular helper cells, and type 17 T helper cells (**Figure 7B**). Furthermore, all identified hub genes exhibited positive correlations with the abundance of immune cells in DM (**Figure 7C**), while the statistical correlations between the four genes and the majority of immune cell types in IPF were found to be insignificant, with the exception of activated B cells and eosinophils (**Figure 7D**).

### 3.5 Prediction of TFs

**Figure 8:**
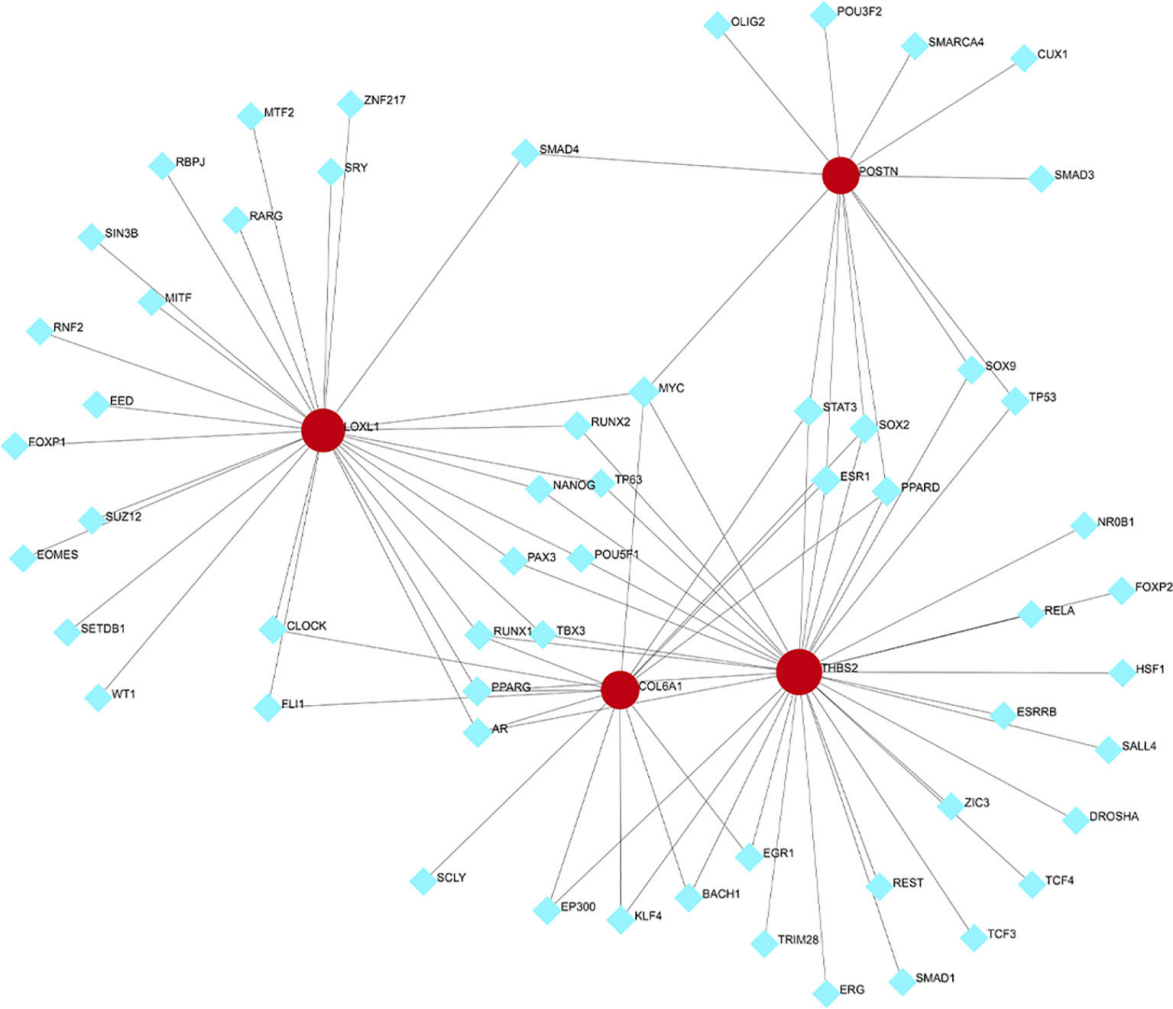
Transcription factors (TFs)- hub genes regulatory network. TFs are represented by the blue diamonds, and hub genes by the red circles.

To predict the TFs interacting with the four hub genes, NetworkAnalyst was used and the resulting TF-gene regulatory network was visualized using Cytoscape. As depicted in **Figure 8**, MYC interact with all four hub genes, suggesting potential regulation of their expression. Nevertheless, additional research is necessary to validate these findings.

## 4 Discussion

The disease progression of DM-ILD is heterogenous. Some patients may experience a progressive fibrosing phenotype characterized by a gradual accumulation of fibrosis on HRCT and a decrease in pulmonary function despite current therapy (4, 20, 25–27). Certain cases can even rapidly progress and pose a life-threatening risk (10, 28, 29). Autoantibodies such as anti-aminoacyl-tRNA synthetase (ARS) and anti-melanoma differentiation-associated gene 5 (MDA5) are related to ILD in DM (30, 31). They can activate innate immune cells, leading to the production of inflammatory cytokines, including interferon alpha (IFNα) and interleukin-18 (IL-18), which can further promote fibrosis and tissue damage in the lungs (2, 32, 33). High levels of IFN-γ and IL-6 have also been observed in DM patients with life-threatening ILD (34). In addition, certain immune cells, such as CD8+ T cells and CD68+ cells, are more prevalent in the lung tissues of DM patients (35, 36). Previous studies have reported some genetic background for the development of ILD in DM, such as gene polymorphisms (STAT4 rs7574865, TNFAIP3 rs2230926 and rs5029939, ANKRD55 rs7731626, and PLCL1 rs6738825 and rs7572733, etc) and differential expressed lncRNAs (37–41). However, there is a lack of large-scale genomic research to explore the common signaling pathways and pathogenesis in DM and IPF.

Comprehensive transcriptomic analyses offer valuable insights into the pathobiology of DM and IPF. Functional enrichment analysis of the WGCNA and DEG datasets consistently reveals the activation of crucial signaling pathways, notably the PI3K-Akt pathway, ECM-receptor interaction and focal adhesion in both DM and IPF. This alignment with our results is seen in the upregulation of PI3K/AKT signaling and cell adhesion molecules in DM (42, 43). Additionally, an elevation in several ECM-degrading enzymes, particularly matrix metalloproteinases (MMPs), is observed in inflammatory myopathies (44). The multifaceted PI3K-Akt pathway significantly contributes to the etiology of IPF, particularly as a critical driver in the direct progression of its pathogenesis. Moreover, the PI3K/AKT pathway exerts substantial regulatory influence over the ECM. This is exemplified by instances such as the initiation of the PI3K/Akt/mTORC1 phosphorylation cascade, facilitating interactions between IPF fibroblasts and collagen-rich matrices. Additionally, focal adhesion kinase (FAK) collaborates with PI3K, impacting cell survival, apoptosis, and cell cycle progression (45). Targeting the PI3K/AKT pathway emerges as a promising avenue for potential IPF therapy (45). In combination, the activation of these cascades may potentially bridge the pathogenic processes of DM and ILD. Therefore, interventions directed at these pathways hold considerable promise for effective DM-ILD management.

Furthermore, four hub genes (*POSTN*, *THBS2*, *COL6A1*, and *LOXL1*) were screened out by intersecting the WGCNA and DEG sets. They were validated to be significantly elevated in DM-ILD patients compared to the control group. *POSTN* encodes the protein periostin, a matricellular protein typically expressed at low levels in normal adult tissue. But its expression is significantly higher at sites of injury or inflammation (46). *POSTN* can activate TGF-β, and it interacts with integrins to facilitate epithelial cell adhesion, migration and mediate tissue remodeling (46–50). It can also interact with other ECM proteins, including collagen I, fibronectin, and tenascin-C, to enhance fibrosis (51). *POSTN* also promotes the differentiation of fibroblasts into myofibroblasts and induces collagen fibrillogenesis and cross-linking (51, 52). A meta-analysis found that elevated *POSTN* expression was associated with interferon signaling in DM (53). In IPF patients, *POSTN* is highly expressed in lung regions undergoing active fibrosis (54). Higher levels of *POSTN* are correlated with fibrotic progression and decreased pulmonary function (55). Thrombospondin-2 (TSP-2), encoded by the *THBS2* gene, belongs to the thrombospondin family. These proteins are known to form trimers and have a distinct N-terminal domain followed by a von Willebrand factor Type C domain (56). TSP-2 is involved in the regulation of cell adhesion, migration, proliferation, as well as angiogenesis and wound healing processes (56). Furthermore, studies have found that TSP-2 is implicated in the pathogenesis of LPS-induced acute respiratory distress syndrome (ARDS) through the activating the PI3K-Akt pathway (57). The *COL6A1* gene, located on chromosome 21, is instrumental in preserving tissue integrity. It is one of the three major genes that encode the collagen VI α-chains, a crucial extracellular matrix protein (58). In line with our study findings, *COL6A1* transcript expression is significantly elevated in DM patients compared with normal controls (59). Mutations in *COL6A1* cause collagen VI-related muscular dystrophy and respiratory dysfunction (60). Simultaneously, while collagen VI assumes a pivotal role in establishing and maintaining lung structure and function, it also exerts a direct influence on lung epithelial cell phenotype *in vitro* (61). IPF patients exhibit heightened *COL6* mRNA and protein deposition, signifying Collagen VI as a pivotal fibrosis driver and disease biomarker (62, 63). *LOXL1* is responsible for encoding the LOXL1 enzyme, which is a copper-dependent monoamine oxidase. This enzyme plays a crucial role in the biogenesis of elastin, specifically by polymerizing tropoelastin monomers into elastin polymers. Besides, *LOXL1* is also involved in the crosslinking of type II collagen (64–66). In DM patients, expression of *LOXL1* exhibits notable elevation (67). Elevated expression of LOXL1 has also been observed in the lungs of mice treated with bleomycin (68). Additionally, both the gene and protein levels of LOXL1 are elevated in IPF tissues compared to other tissues (69). Interestingly, the lungs of mice lacking *LOXL1* were found to have protection against experimental fibrosis following adenoviral (Ad) gene transfer, which facilitated the overexpression of TGF-β1 (AdTGF-β1). The absence of *LOXL1* prevents the buildup of insoluble, cross-linking collagens in the lungs, thereby limiting lung stiffness following AdTGF-β1 treatment. These finding suggest that cross-linking enzymes like *LOXL1* could be potential targets for therapeutic drug development in IPF (70). Taken together, the elevated levels of *POSTN*, *THBS2*, *COL6A1*, and *LOXL1* may contribute to the development of pulmonary fibrosis, similar to what is seen in IPF, thus connecting fibrosis to inflammation in DM-ILD.

When looking into the immune infiltration microenvironment of DM and IPF, different patterns were seen. Various immune cells were activated in DM, leading to a series of immune responses, including cellular and humoral responses. On the other hand, IPF demonstrated restricted immune cell activation compared to DM. This was evident by the predominant activation of memory B cells, monocytes and Th2 cells in IPF suggesting a more pronounced humoral response. This finding aligns with the discovery that a subset of patients with IPF has autoantibodies targeting various intracellular components (71, 72). Additionally, B cells in IPF patients show enhanced antigen differentiation and higher proportions of plasma blasts compared to those found in normal controls (73). Moreover, all four genes were positively correlated with immune cells abundance in DM but not in IPF. However, it is important to note that this correlation does not necessarily indicate a causal relationship between these genes and immune responses in DM. In the context of DM, the robust activation of immune responses and concomitant necrosis of muscle fibers contribute to tissue repair and regeneration. As a result, four key genes that play a crucial role in tissue remodeling and fibrosis exhibit a positive correlation with the infiltration of immune cells in DM. Conversely, IPF, a disease primarily characterized by fibrosis, has minimal immune response. Therefore, no correlation between these genes and immune response has been observed.

In conclusion, we found that there are shared molecular mechanisms underlying the development of DM and IPF. Bioinformatics analysis revealed several biological pathways are enriched, including ECM organization, collagen fibril organization, and regulation of cell adhesion, suggesting dysregulation of these pathways may contribute to the development of ILD in DM patients. There are still a few limitations in our study, including a small sample size for validation and a lack of subgroup analysis based on the myositis specific antibodies. Besides, since no samples from IPF patients were available, a comparative assessment of the transcriptional levels of the four genes between DM-ILD and IPF cohorts could not be performed. Nevertheless, the results of our study provide new insights into the molecular mechanisms underlying the development of DM-ILD and may help to identify potential therapeutic targets for the disease. However, further research is still needed to validate these findings and to explore the functional roles of the identified genes and pathways in the pathogenesis of DM-ILD.

## Data availability statement

The datasets presented in this study can be found in online repositories. The names of the repository/repositories and accession number(s) can be found below: https://www.ncbi.nlm.nih.gov/geo/, GSE143323, GSE150910, GSE134692 and GSE128470.

## Ethics statement

The studies involving humans were approved by Ethics Committee of Sichuan Provincial People’s Hospital. The studies were conducted in accordance with the local legislation and institutional requirements. The participants provided their written informed consent to participate in this study.

## Author contributions

LZ: Conceptualization, Data curation, Formal Analysis, Funding acquisition, Investigation, Methodology, Project administration, Writing – original draft, Writing – review & editing. YT: Data curation, Investigation, Methodology, Validation, Writing – original draft, Writing – review & editing. YZ: Data curation, Project administration, Validation, Writing – review & editing. LYu: Data curation, Project administration, Validation, Writing – review & editing. GM: Data curation, Writing – review & editing. XY: Data curation, Writing – review & editing. LYa: Data curation, Writing – review & editing. KC: Data curation, Investigation, Methodology, Project administration, Software, Writing – review & editing. QZ: Conceptualization, Formal Analysis, Investigation, Methodology, Project administration, Supervision, Validation, Writing – original draft, Writing – review & editing.
