# Supplementary material for: The molecular mechanism underlying dermatomyositis related interstitial lung disease: evidence from bioinformatic analysis and in vivo validation
Source: Front Immunol. 2023 Oct 19;14:1288098. doi: 10.3389/fimmu.2023.1288098 (PMC10622801; doi:10.3389/fimmu.2023.1288098)
Supplement: Supplementary file 1 [file DataSheet_1.docx]

# Supplementary table 1. Detailed information of DM and IPF databases.

|  | GSE Number | Platform | Samples | Organism | Source types | Disease |
| --- | --- | --- | --- | --- | --- | --- |
| 1 | GSE143323 | GPL21290 | 39 DM and 20 controls | Homo sapiens | Muscle | DM |
| 2 | GSE150910 | GPL24676 | 103 IPF and 103 controls | Homo sapiens | Lung | IPF |
| 3 | GSE128470 | GPL96 | 12 DM and 12 controls | Homo sapiens | Muscle | DM |
| 4 | GSE134692 | GPL16791 | 46 IPF and 26 controls | Homo sapiens | Lung | IPF |

DM, dermatomyositis; IPF, idiopathic pulmonary fibrosis.
