# Supplementary material for: The molecular mechanism underlying dermatomyositis related interstitial lung disease: evidence from bioinformatic analysis and in vivo validation
Source: Front Immunol. 2023 Oct 19;14:1288098. doi: 10.3389/fimmu.2023.1288098 (PMC10622801; doi:10.3389/fimmu.2023.1288098)
Supplement: Supplementary file 2 [file DataSheet_2.pdf]

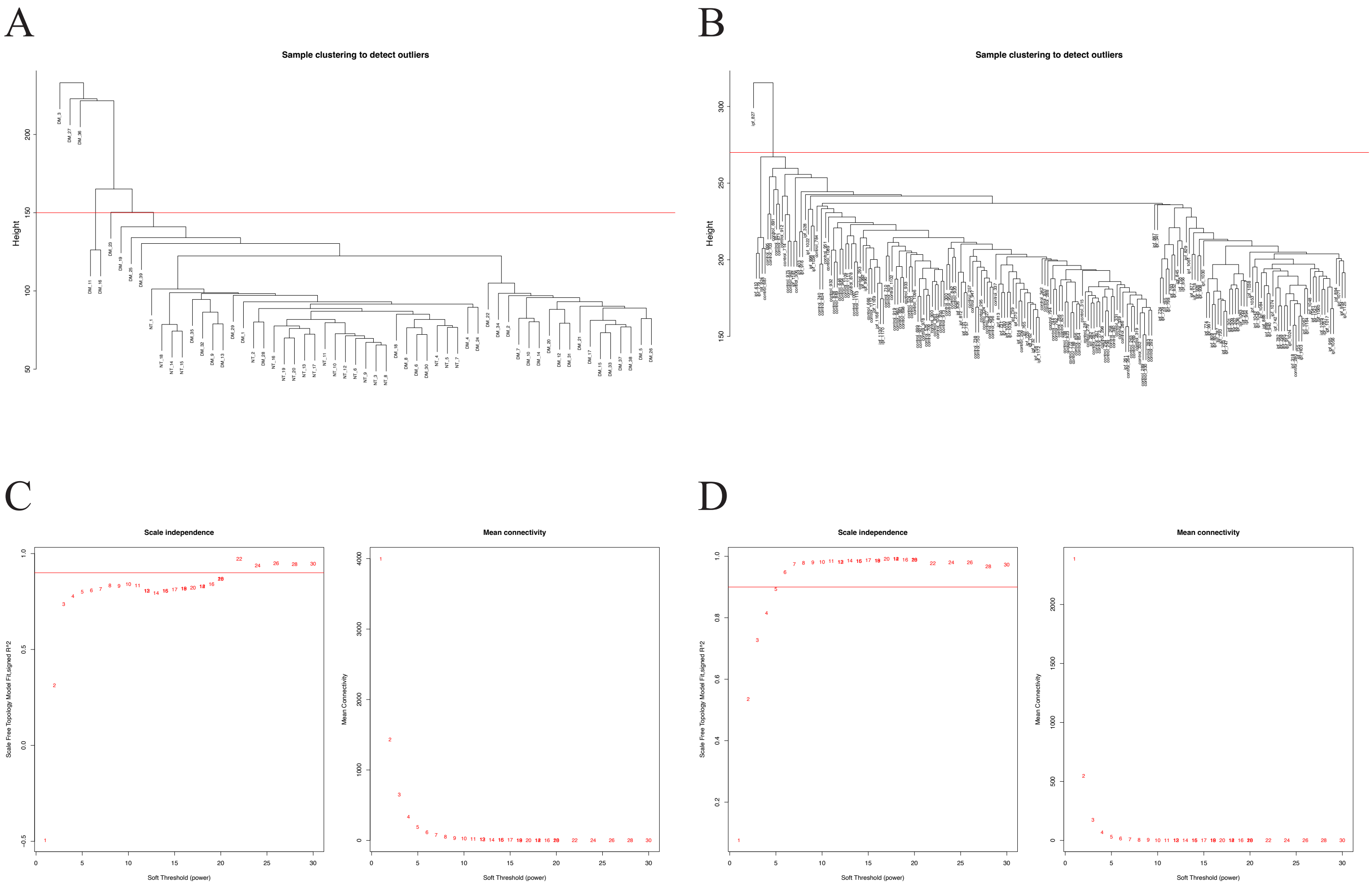

Supplementary File 2: (A-B) Sample clustering to detect outliers in GSE143323 (A) and GSE150910 (B) datasets. (C-D) The determination of soft thresholding power in GSE143323 (C) and GSE150910 (D) datasets.
